# Piperacillin–tazobactam-associated thrombocytopenia with negative antiplatelet antibody testing: a case report and literature review

**DOI:** 10.3389/fmed.2026.1775491

**Published:** 2026-03-11

**Authors:** Pengcheng Tian, Ming Fan, Chaolin Huang

**Affiliations:** 1 Wuhan Jinyintan Hospital, Tongji Medical College of Huazhong University of Science and Technology, Wuhan, China; 2 Hubei Clinical Research Center for Infectious Diseases, Wuhan, China; 3 Wuhan Research Center for Communicable Disease Diagnosis and Treatment, Chinese Academy of Medical Sciences, Wuhan, China; 4 Joint Laboratory of Infectious Diseases and Health, Wuhan Institute of Virology and Wuhan Jinyintan Hospital, Chinese Academy of Sciences, Wuhan, China; 5 Institute of Hematology, Union Hospital, Tongji Medical College, Huazhong University of Science and Technology, Wuhan, China

**Keywords:** anti-platelet antibodies, case report, drug-induced immune thrombocytopenia (DIIT), piperacillin-tazobactam, thrombocytopenia

## Abstract

**Background:**

Piperacillin–tazobactam is a widely used antibiotic. Although rare, thrombocytopenia related to piperacillin–tazobactam can be clinically significant. Drug-induced immune thrombocytopenia (DIIT) is difficult to distinguish from thrombocytopenia related to critical illness, infection, disseminated intravascular coagulation (DIC), or heparin-induced thrombocytopenia (HIT). We report a case of piperacillin–tazobactam-associated thrombocytopenia and review the literature to emphasize that negative antiplatelet antibody testing does not exclude this diagnosis.

**Case presentation:**

We report a 57-year-old woman with severe trauma and pulmonary infection who developed abrupt, severe thrombocytopenia after initiating piperacillin–tazobactam. The platelet count recovered rapidly after piperacillin–tazobactam was withdrawn. Coagulation parameters did not support overt DIC as the primary cause, and HIT was considered unlikely (4Ts score ≤3; no thrombosis on serial bedside ultrasonography). A Naranjo Adverse Drug Reaction Probability Scale score of 5 indicated a probable adverse drug reaction. Flow cytometric testing for anti-platelet antibodies was negative.

**Review:**

We identified 23 eligible reports. All patients were considered immune-mediated. Antiplatelet antibody testing was not performed on many patients; importantly, piperacillin–tazobactam-associated immune thrombocytopenia has also been suspected in cases with negative antibody results.

**Conclusion:**

This case and the reviewed literature highlight that negative platelet antibody testing does not exclude piperacillin–tazobactam-associated DIIT. A structured diagnostic approach and close platelet monitoring are warranted when DIIT is suspected in critically ill patients. Clinical evaluation and temporal association remain critical for diagnosis.

## 1 Background

Pulmonary infections are a common complication in hospitalized patients, especially those requiring prolonged mechanical ventilation. The main treatment for pulmonary infections is the systematic use of antibiotics. Piperacillin–tazobactam, a commonly used ureidopenicillin/beta-lactamase inhibitor combination, treats infections caused by beta-lactamase-producing bacteria (1, 2). It is also a form of empiric therapy for certain conditions, such as fever or neutropenia (3). Even in some special cases, piperacillin–tazobactam can effectively replace carbapenems, thus reducing the use of broad-spectrum antibiotics (4). Although generally well tolerated, adverse reactions such as diarrhea, rash (erythema), and kidney injury have been reported (5, 6).Other rare adverse reactions include allergic shock, exfoliative dermatitis, and nervous system abnormalities (7). Thrombocytopenia is rare but clinically important.

Drug-induced immune thrombocytopenia (DITP) is usually characterized by an abrupt platelet fall and rapid recovery after drug withdrawal. The patient was diagnosed with thrombocytopenia when the platelet count was below 100 × 10^9^/L. The main complication of thrombocytopenia is bleeding, including skin, mucosa, and even gastrointestinal or intracranial bleeding (8). A variety of antibiotics, such as linezolid, can cause thrombocytopenia (9), and piperacillin–tazobactam has previously been reported to cause thrombocytopenia (10). However, little is known about the pathogenesis of thrombocytopenia caused by piperacillin–tazobactam. At present, immune thrombocytopenia has been most frequently mentioned. Antiplatelet antibodies are often considered supportive but are not consistently detected. We present a case and literature review highlighting that negative antiplatelet antibody testing does not rule out piperacillin–tazobactam-associated thrombocytopenia.

## 2 Case reports

**Figure 1:**
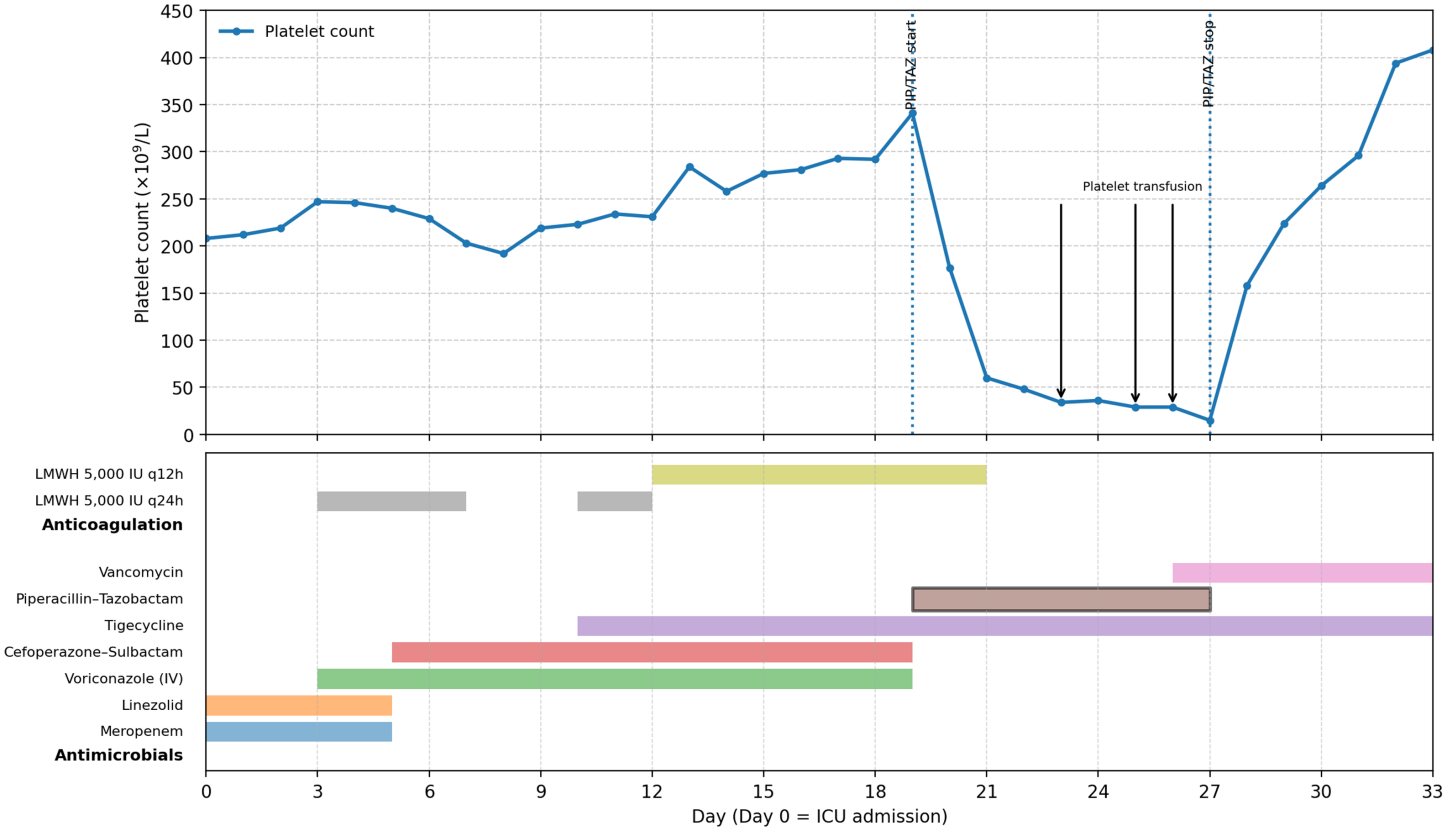
PLT level during the piperacillin–tazobactam treatment. Platelet count decreased immediately after piperacillin–tazobactam initiation and increased after stopping the use of piperacillin–tazobactam.

A 57-year-old woman was hospitalized after trauma with multiple fractures and underwent surgical management. She was subsequently transferred to the intensive care unit (ICU) due to respiratory failure and pulmonary infection, where she received broad-spectrum antibiotics. Multiple antibiotic adjustments were made based on her clinical course and culture results (Table 1). After piperacillin–tazobactam initiation (day 19), serial complete blood counts showed an abrupt and progressive decline in platelet counts. Platelet counts were 341 × 10^9^/L on day 19, decreased to 177 × 10^9^/L on day 20, 60 × 10^9^/L on day 21, and reached a nadir of 15 × 10^9^/L on day 27 (Figure 1).

**Table 1:** Timeline of ICU course, antimicrobial exposure, microbiology, and laboratory trends (Day 0–33).

Day/date	Key events/microbiology	Antimicrobials (dose/route)	PLT	WBC	Hb	Coagulation (PT/aPTT/Fib/D-dimer)	Concomitant meds/interventions	Notes
Day 0	ICU admission	Meropenem 500 mg q8h IV + linezolid 0.6 g q12h IV	208	6.7	74	15.2/40.0/3.42/NA	—	Baseline labs
Day 1	—	Meropenem + linezolid continued	212	4.3	72	15.9/37.0/3.13/NA	—	
Day 2	Sputum culture: *Aspergillus fumigatus*	Meropenem + linezolid continued	219	3.1	77	NA	—	Culture result obtained
Day 3	—	Voriconazole 200 mg q12h IV added (to Day 19) + meropenem + linezolid	247	4.8	84	NA	LMWH 5000 IU SC daily started (Day 3–7)	
Day 4	—	Meropenem + linezolid + voriconazole	246	5.47	85	NA	LMWH 5000 IU SC daily	
Day 5	Sputum culture: abundant *A. baumannii*	Meropenem and Linezolid stopped; cefoperazone–sulbactam 2.0 g q6h IV started (to Day 19) + voriconazole	240	6.2	88	NA	LMWH 5000 IU SC daily	
Day 6	—	Cefoperazone–sulbactam + voriconazole	229	7.79	84	NA	LMWH 5000 IU SC daily	
Day 7	—	Cefoperazone–sulbactam + voriconazole	203	8.03	71	NA	LMWH 5000 IU SC daily (last day)	
Day 8	—	Cefoperazone–sulbactam + voriconazole	192	6.07	63	NA	—	
Day 9	Sputum culture: abundant CRAB	Cefoperazone–sulbactam + voriconazole	219	6.41	86	14.7/43.8/4.24/NA	—	
Day 10	—	Tigecycline loading 100 mg IV, then 50 mg q12h IV (to Day 33) + cefoperazone–sulbactam + voriconazole	223	7.92	85	NA	LMWH 5000 IU SC daily (Day 10–12)	
Day 11	—	Same as above	234	10.31	82	NA	LMWH 5000 IU SC daily	
Day 12	—	Same as above	231	10.1	86	14.8/42.9/3.85/NA	LMWH changed to 5,000 IU SC q12h (Day 12–20)	
Day 13	—	Same as above	284	6.93	83	NA	LMWH 5000 IU SC q12h	
Day 14	—	Same as above	258	12.0	80	NA	LMWH 5000 IU SC q12h	
Day 15	—	Same as above	277	11.0	81	NA	LMWH 5000 IU SC q12h	
Day 16	—	Same as above	281	12.58	79	NA	LMWH 5000 IU SC q12h	
Day 17	—	Same as above	293	13.43	86	14.4/42.4/2.89/NA	LMWH 5000 IU SC q12h	
Day 18	Sputum culture: *Enterobacter aerogenes*	Cefoperazone–sulbactam + tigecycline + voriconazole	292	14.3	77	14.7/40.1/2.79/NA	LMWH 5000 IU SC q12h	
Day 19	Susceptibility-guided adjustment	Piperacillin–tazobactam 4.5 g q8h IV added; cefoperazone–sulbactam stopped; voriconazole stopped; tigecycline continued	**341**	11.64	79	NA	LMWH 5000 IU SC q12h	Start of suspected culprit exposure window
Day 20	—	Piperacillin–tazobactam + tigecycline	177	9.03	86	NA	LMWH ended (last day)	
Day 21	—	Piperacillin–tazobactam + tigecycline	60	8.82	84	NA	—	Marked thrombocytopenia begins
Day 22	—	Piperacillin–tazobactam + tigecycline	48	11.92	97	NA	—	
Day 23	—	Piperacillin–tazobactam + tigecycline	34	13.5	94	15.4/38.2/2.14/NA	Platelet transfusion: 1 therapeutic dose	
Day 24	—	Piperacillin–tazobactam + tigecycline	36	9.70	84	NA	—	
Day 25	Sputum culture: small amount *S. aureus* + small amount *A. baumannii*	Piperacillin–tazobactam + tigecycline continued	29	8.46	91	14.2/35.9/1.86/1.58	Platelet transfusion: 1 therapeutic dose	Flow cytometry for anti-platelet antibodies: negative
Day 26	—	Vancomycin 0.5 g q6h IV added (to Day 33) + piperacillin–tazobactam + tigecycline	29	10.17	85	NA	Platelet transfusion: 2 therapeutic doses	
Day 27	Platelets continued to decline during exposure	Piperacillin–tazobactam discontinued; Vancomycin + tigecycline continued	**15**	8.21	85	NA	—	Nadir PLT = 15
Day 28	+24 h after discontinuation	Vancomycin + tigecycline continued	158	9.63	89	14.2/35.2/2.60/1.64	—	Rapid recovery within 24 h
Day 29	+48 h after discontinuation	Vancomycin + tigecycline continued	224	9.4	89	13.7/34.9/2.29/NA	—	
Day 30	+72 h after discontinuation	Vancomycin + tigecycline continued	264	8.87	92	NA	—	
Day 31	—	Vancomycin + tigecycline continued	296	7.60	89	NA	—	
Day 32	—	Vancomycin + tigecycline continued	394	9.29	97	NA	—	
Day 33	Discharge	Vancomycin and tigecycline discontinued at discharge	408	9.44	95	NA	—	

Units: PLT/WBC (×10^9^/L); Hb (g/L); PT/aPTT (seconds); fibrinogen (g/L); D-dimer (mg/L FEU). The institutional reference ranges: PT 11.0–16.0 s, aPTT 28.0–43.5 s, fibrinogen 2.0–4.0 g/L, D-dimer <0.5 mg/L FEU; platelet count 125–350 × 10^9^/L; hemoglobin 115–150 g/L; and WBC 3.5–9.5 × 10^9^/L. Day 0, ICU admission. “NA”, not available/tested.

During this period, no clinically overt bleeding was observed (no petechiae, mucosal bleeding, hematuria, or gastrointestinal bleeding). Daily bedside ultrasonography demonstrated no evidence of thrombosis in the extremities or in the internal jugular veins.

Coagulation parameters did not support the presence of overt disseminated intravascular coagulation as the primary cause of thrombocytopenia. Heparin-induced thrombocytopenia (HIT) was considered unlikely. In addition, flow cytometric testing for anti-platelet antibodies was negative on day 25. The patient’s clinical presentation does not support consideration of thrombotic thrombocytopenic purpura. Infection indicators were stable during the platelet decline. White blood cell counts were only mildly elevated, and procalcitonin remained within the reference range (<0.5 ng/mL) (Table 2). Prolonged antibiotic therapy was continued because of the recent severe respiratory infection and the culture-documented Enterobacter infection, despite normalized inflammatory markers.

**Table 2:** Procalcitonin (PCT) levels during the piperacillin–tazobactam treatment.

Time	PCT reference range (<0.5 ng/mL)
12 July	0.05 ng/mL
17 July	0.22 ng/mL
21 July	0.06 ng/mL

After review of the literature, piperacillin–tazobactam-associated thrombocytopenia was suspected. Piperacillin–tazobactam was discontinued on day 27. Platelet counts increased rapidly thereafter. Using the Naranjo Adverse Drug Reaction Probability Scale, the score was 5 (probable).

## 3 Methods

We searched PubMed for English-language reports using MeSH terms and keywords (“piperacillin” OR “piperacillin–tazobactam” OR “tazobactam”) AND “thrombocytopenia.” We included case reports, case series, and observational studies reporting patient-level platelet outcomes. Reference lists of included articles were also screened.

## 4 Results

**Figure 2:**
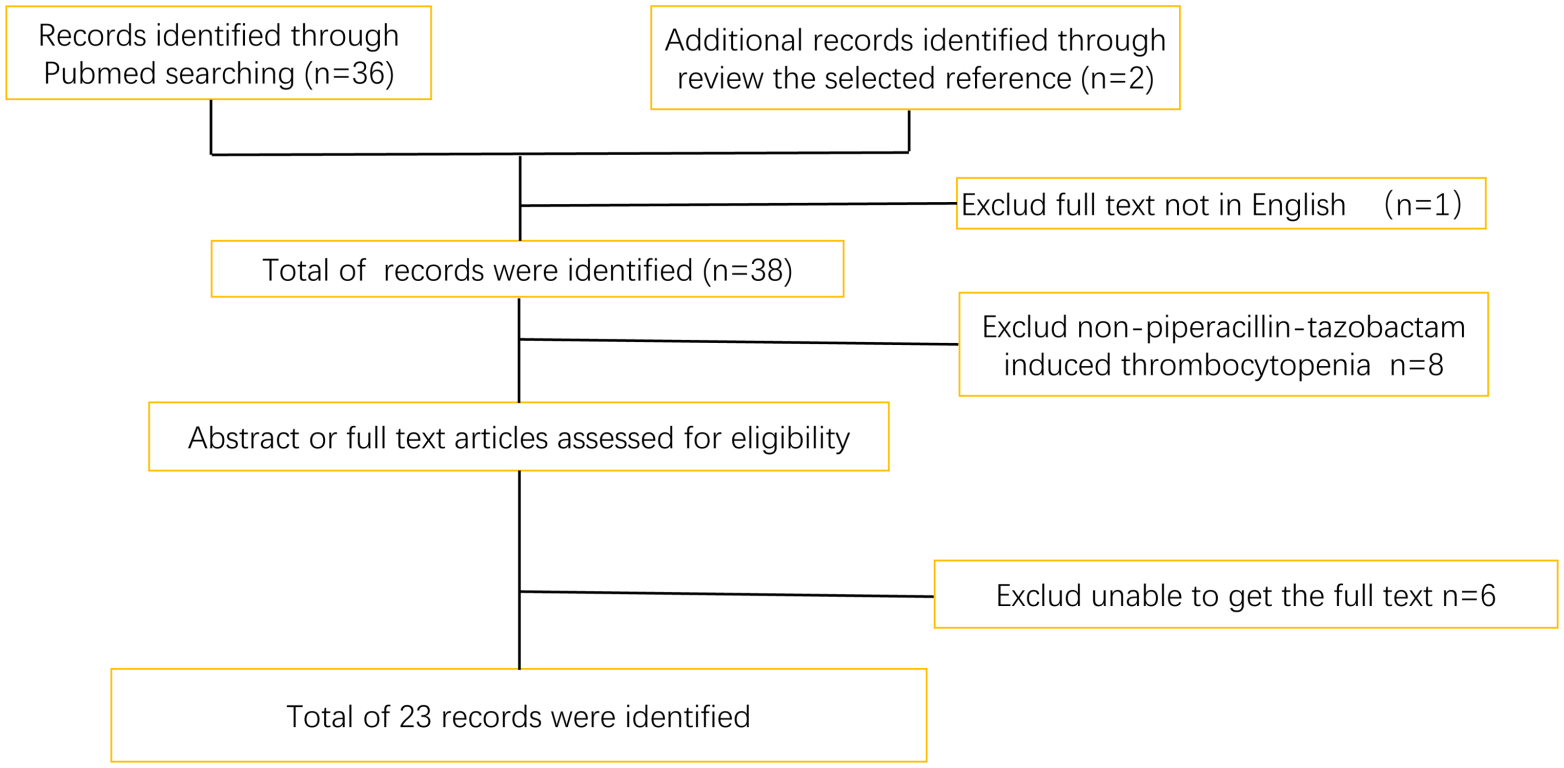
Flow chart describes the selection process of the references.

We identified 23 records with titles/abstracts related to piperacillin–tazobactam and thrombocytopenia. After full-text review, one comparative study was excluded because it lacked patient-specific information (Figure 2).

Overall, 61 patients with piperacillin–tazobactam-induced thrombocytopenia were included (Table 3). Thrombocytopenia typically occurs approximately 10 days after starting therapy; some cases occur on re-exposure. In most reports, piperacillin–tazobactam was stopped or replaced promptly, and platelet recovery followed within several days. Some patients received platelet transfusions, corticosteroids, or intravenous immunoglobulin.

**Table 3:** Literature about piperacillin–tazobactam-related thrombocytopenia.

Authors	Year	Type of article	Journal	Number of patients	Anti-platelet antibodies	Treatments
Al-Sardi et al. (17)	2021	Case report	Cureus	1	Not measured	Drug withdrawal and platelets
Lv et al. (18)	2021	Case report	European Journal of Hospital Pharmacy	1	Not measured	Drug withdrawal
Chen et al. (19)	2020	Correspondence	British Journal of Haematology	3	Positive (flow cytometry)	Drug withdrawal
Chen et al. (20)	2019	Case report	BMJ Case Reports	1	Not measured	Drug withdrawal, platelets, and dexamethasone
Beaulieu et al. (21)	2019	Article	Journal of Thrombosis and Thrombolysis	1	Negative (flow cytometry)	Drug withdrawal, dexamethasone and immunoglobulin
Alzahrani et al. (22)	2018	Review	Platelets	2	Not measured	Drug withdrawal, corticosteroids and immunoglobulin
Patel et al. (23)	2017	Case report	Transplantation Proceedings	1	Positive (not mentioned)	Drug withdrawal
Boyce et al. (24)	2016	Case report	Journal of Clinical Pharmacy and Therapeutics	1	Not measured	Platelets and corticosteroids
Chen et al. (7)	2016	Case report	Experimental and Therapeutic Medicine	1	Negative (ELISA)	Drug withdrawal
Nguyen et al. (25)	2015	Case report	Pharmacotherapy	1	Not measured	Drug withdrawal and platelet
Bose et al. (26)	2015	Case report	Journal of Clinical Anesthesia	1	Positive (flow cytometry)	Drug withdrawal, platelet, and immunoglobulin
Hron et al. (27)	2013	Article	Upsala Journal of Medical Sciences	1	Positive (not mentioned)	Drug withdrawal
Uzun et al. (28)	2013	Case report	Scandinavian Journal of Infectious Diseases	1	Not measured	Drug withdrawal
Lin et al. (29)	2012	Case report	Hemodialysis International	1	Not measured	Drug withdrawal and platelet
Anand et al. (13)	2011	Case report	Platelets	1	Positive (flow cytometry)	Drug withdrawal
Reichardt et al. (12)	1999	Brief reports	Infection	38	Not measured	Drug withdrawal
Rousan et al. (30)	2010	Article	American Journal of Hematology	1	Not measured	Drug withdrawal
He et al. (31)	2023	Case report	Journal of Medical Internet Research	1	Positive (not mentioned)	Drug withdrawal
He et al. (16)	2013	Case Report	Scandinavian Journal of Infectious Diseases	1	Not measured	Drug withdrawal
He et al. (32)	2022	Letter	Surgical Infections	1	Not measured	Drug withdrawal, platelet, immunoglobulin, and glucocorticoid
Yata et al. (33)	2000	Case report	Annals of Hematology	1	Not measured	Plasma infusion

Of these patients, the majority (51) were not tested for anti-platelet antibodies. Drug-dependent immune mechanisms have been supported by reports with positive antiplatelet antibody findings. Notably, immune-mediated thrombocytopenia has also been suspected in patients with negative antibody results, likely reflecting assay limitations and the timing of testing.

## 5 Discussion

Piperacillin–tazobactam is commonly used in the ICU, but thrombocytopenia caused by piperacillin–tazobactam is rare (7). Drug-induced immune thrombocytopenia (DIIT) is an important and potentially life-threatening adverse drug reaction. In critically ill patients, thrombocytopenia is common and frequently multifactorial, including sepsis-related consumption, disseminated intravascular coagulation (DIC), heparin-induced thrombocytopenia (HIT), massive transfusion, and drug-related bone marrow suppression. In this case, the patient developed abrupt, severe thrombocytopenia shortly after initiation of piperacillin–tazobactam, with a nadir of 15 × 10^9/L and rapid recovery within 24 h after drug discontinuation, which strongly supports DIIT as the most likely etiology.

DIC was considered but was not supported as the primary cause of thrombocytopenia. During the period of severe thrombocytopenia, PT and aPTT were within or near institutional reference ranges, with only mild transient decreases in fibrinogen (e.g., 1.86 g/L on day 25) and elevated D-dimer (e.g., 1.58 mg/L FEU on day 25). The overall pattern did not fulfill typical criteria for overt DIC and did not parallel the dramatic platelet recovery observed immediately after piperacillin–tazobactam discontinuation.

HIT was also considered unlikely: The patient received low-molecular-weight heparin (LMWH) 5,000 IU subcutaneously once daily from Day 3 to Day 7, and from days 10 to 12, followed by 5,000 IU subcutaneously every 12 h from days 12 to 20. For the thrombocytopenia component of the 4Ts score, the platelet count decreased by >50% with a recorded nadir of 15 × 10^9^/L. However, platelet transfusion was administered the evening prior to this measurement, which may have influenced the observed nadir. As a nadir <10 × 10^9^/L would correspond to 0 points in the 4Ts scoring system, this component was conservatively assigned no more than 1 point given that cumulative heparin exposure spanned more than 10 days; Thrombosis: 0 points due to negative daily bedside ultrasonography; and other causes: 1 point as drug-induced thrombocytopenia was considered possible. The 4Ts score was ≤3 (low probability) in the setting of intermittent LMWH exposure, absence of thrombosis on serial ultrasonography, and the presence of a plausible alternative cause (suspected DIIT). Therefore, PF4/heparin ELISA and functional assays were not pursued.

Drug-induced thrombocytopenia (DITP, including DIIT) is a kind of disease in which the peripheral blood platelet count is lower than the normal standard due to the use of related drugs, resulting in thrombocytopenia-related complications (11). Diagnostic criteria for DITP can be summarized as follows: (1) exposure to a drug known to cause thrombocytopenia, (2) normal platelet count before exposure, with a fall after exposure and recovery after drug withdrawal, (3) other causes of thrombocytopenia reasonably excluded, and (4) recurrence on re-exposure. Meeting all four criteria supports a definite diagnosis; meeting three supports a probable diagnosis. Because re-exposure was not performed, criterion (4) could not be assessed. Therefore, it is considered that thrombocytopenia may be caused by piperacillin–tazobactam. The expected time course of DITP typically includes a platelet fall within approximately 5–10 days of first exposure and a rapid recovery within several days after discontinuation. Rapid recovery in this case was consistent with this pattern.

Several studies have reported the phenomenon of thrombocytopenia in patients receiving piperacillin–tazobactam therapy. A retrospective study of 38 children with cystic fibrosis suggested that thrombocytopenia was related to piperacillin–tazobactam and was time- and dose-dependent, occurred after 11 to 15 days of treatment (12). Similarly, piperacillin–tazobactam could cause thrombocytopenia in hospital patients, especially in acutely ill or elderly (13). And piperacillin–tazobactam-related thrombocytopenia was associated with 30-day all-cause mortality in patients with *Pseudomonas aeruginosa* bacteremia (14). However, analysis of a randomized controlled trial demonstrated that single-use piperacillin–tazobactam may not contribute to a lower platelet count in critically ill patients (15).

Published studies reporting the incidence of thrombocytopenia in patients treated with piperacillin–tazobactam are summarized in Table 3. Piperacillin–tazobactam-induced thrombocytopenia is well documented in these articles, although many of them are case reports.

The mechanisms of DITP can broadly be categorized as follows. First, thrombocytopenia due to bone marrow suppression may occur with certain agents (e.g., linezolid), which can impair megakaryopoiesis or be directly toxic to megakaryocytes. This mechanism often results in thrombocytopenia accompanied by other cytopenias (i.e., pancytopenia). Bone marrow suppression can sometimes present with isolated thrombocytopenia; however, it typically shows a slower recovery after stopping the culprit drug. Piperacillin–tazobactam has also been reported to be associated with bone marrow suppression and related hematologic adverse events (16). However, the patient developed abrupt and severe thrombocytopenia, followed by rapid platelet recovery after discontinuation of piperacillin–tazobactam, which is less consistent with the bone marrow suppression mechanism.

Second, certain drugs may reduce platelet counts through non-immune mechanisms, such as direct platelet toxicity or impaired platelet production. Although the precise pathways are not fully elucidated and the decline is often modest in many reported settings, our patient developed abrupt and severe thrombocytopenia. Taken together, the clinical pattern in this case does not strongly support a predominantly non-immune mechanism.

Third, drug-induced immune thrombocytopenia (DIIT) occurs when a drug triggers the formation of platelet-reactive, often drug-dependent, antibodies that accelerate platelet clearance and may cause severe isolated thrombocytopenia without concomitant cytopenias. Although flow cytometric testing for anti-platelet antibodies was negative in our patient, we considered piperacillin–tazobactam–associated DIIT the most plausible explanation, given the clinical time course. In our literature review, several reports suggested that anti-platelet antibody testing may aid diagnosis and differential diagnosis; however, many published cases of suspected piperacillin–tazobactam-induced DIIT reported negative or untested antibody results, indicating that a negative test does not exclude DIIT. This may reflect assay limitations and the timing of sample collection relative to antibody generation and persistence. Therefore, further studies are needed to clarify the molecular pathogenesis of piperacillin–tazobactam-associated thrombocytopenia and to define the diagnostic performance and clinical role of anti-platelet antibody testing.

The primary management of suspected DITP is immediate withdrawal of the offending drug. Discontinuation of piperacillin and tazobactam-induced thrombocytopenia, both in our case and in the literature, is clearly effective and cost-effective. Although some clinical cases have been treated with immunoglobulins, hormones, etc., the specific effect is not very clear, because many cases have stopped taking the drug before use. The effect of platelet infusion before stopping the drug also appears uncertain.

A key feature of this case is that flow cytometric testing for anti-platelet antibodies was negative (Day 25). Many assays performed in routine practice detect platelet-reactive antibodies under non-drug-dependent conditions, whereas DIIT is often mediated by drug-dependent antibodies that require the presence of the implicated drug (or metabolites) to bind platelet glycoproteins *in vitro*. In addition, assay performance may vary across laboratories, and sensitivity can be affected by the timing of specimen collection relative to drug exposure and immune complex formation. Therefore, a negative platelet antibody test—particularly when it is not a validated drug-dependent antibody assay—does not rule out DIIT when the clinical time course is strongly suggestive. Given the low sensitivity and limited availability of antibody testing, routine testing in all patients is not recommended. Instead, platelet monitoring should be guided by the clinical context and duration of therapy, with increased vigilance during prolonged treatment or when thrombocytopenia is suspected. We have clarified that outpatient use should be restricted to settings where appropriate clinical follow-up and laboratory monitoring (e.g., outpatient parenteral antimicrobial therapy programs) can be reliably ensured.

## 6 Confounders and limitations

This case has several limitations. First, the patient was critically ill with severe trauma, multiple surgeries, and ongoing pulmonary infections, all of which can contribute to thrombocytopenia and complicate causal inference. Second, platelet transfusions were administered during the period of severe thrombocytopenia (including two therapeutic doses the evening before the nadir measurement), which may have influenced observed platelet trends and limited precise quantification of the spontaneous nadir. Third, PF4/heparin ELISA and functional testing were not performed; however, the low 4Ts score and absence of thrombosis made HIT less likely. Finally, drug-dependent platelet antibody testing specific to piperacillin–tazobactam was not available, limiting mechanistic confirmation. Despite these constraints, the reproducible pattern of rapid platelet recovery after drug discontinuation remains highly characteristic of DIIT.

## 7 Conclusion

This case underscores several practical points for clinicians. In patients receiving piperacillin–tazobactam—especially in the ICU where alternative causes of thrombocytopenia are common—abrupt, severe thrombocytopenia should prompt consideration of DIIT, even when platelet antibody testing is negative. When DIIT is suspected, prompt discontinuation of the suspected agent and close monitoring of platelet counts are warranted, while concurrently evaluating for other high-risk causes such as DIC and HIT. In most cases, platelets recover after discontinuation of piperacillin–tazobactam. In selected severe cases, supportive care (including transfusion, glucocorticoids, immunoglobulin, and adjunctive therapies based on bleeding risk) may be appropriate. In conclusion, although thrombocytopenia caused by piperacillin–tazobactam is a reversible clinical condition, it should be of great concern to clinicians.

## Data Availability

The original contributions presented in the study are included in the article/supplementary material, further inquiries can be directed to the corresponding author.
